# An Emergency Distribution of £250,000

**Published:** 1920-06-26

**Authors:** 


### KING EDWARD'S HOSPITAL FUND FOR LONDON.

### An Emergency Distribution of £250,000.

A Special Meeting of the President and General Council of King Edward's Hospital Fund for London was held at St. James's Palace on June 22, the Earl of Donoughmore, K.P. (Member of the President's Powers Committee), being in the chair.

Sir Frederick Fry (Hon. Secretary) read the order of appointment of the following new members of the General Council, as follows: The Viscountess Harcourt, the Viscountess Northcliffe, the Lady Ampthill, Lady Hall.

### The Present Hospital Crisis.

Lord Donoughmore, moving the resolution "That an emergency distribution of £250,000 be made out of the available general funds to assist the London hospitals, having regard to their pressing needs," said: I ventured to remind you at the last meeting of the Council that this Fund was originally established by his late Majesty King Edward VII. to deal with a hospital crisis which was then very much in existence. Since our last meeting the executive officers of the Fund have been actively exploring the present position and considering the various alternatives, both independently and also in consultation with others concerned in the question of rendering help to those responsible for our voluntary hospitals. It soon became quite evident that it was a problem which must be divided into two different parts. There is the problem of the present debt of the hospitals, and there is the problem of their future maintenance. Both problems arise from the same cause. They arise partly from the difficulties which the hospitals got into during the war, and partly to the loss they have suffered since the war in the subscribing power of their friends and the difficulties of those anxious and willing to help them by continuing or increasing their subscriptions. The problem of the organisation necessary to obtain fresh additions of income will take time. But the problem of the present debt will not wait: it has got to be dealt with, and, if it is not dealt with, the double burden that the hospitals are under may prove to be too great to be borne. It is a problem that requires the co-operation of all the agencies concerned; and, as I said, our principal executive officers here have been taking part in general discussions, exploring the ground. They have received valuable information from the newly formed London Committee of the British Hospitals Association. The ideal, of course, is to secure the advantages of co-operation with those who are interested in the work without at the same time losing the established connections and organisation of the existing institutions, and their individual initative and originality. I need not say that we especially welcome the action of the Joint Committee of the British Red Cross Society and the Order of Saint John of Jerusalem, who are now applying the great organising power which they had developed before the war, and especially during the war, to the assistance of the voluntary hospitals.

It must be remembered that the voluntary hospital problem is one for the whole country; it is not merely for London. We are concerned, like the Metropolitan Sunday Fund and the Saturday Fund, with the London hospitals alone; but we naturally are very much indebted and grateful for the efforts that are made outside London by the Joint Committee and those who work with them. The concern of the King's Fund being, however, with the London hospitals, we now propose this emergency distribution of a quarter of a million out of the available general funds to assist the London hospitals in their pressing needs. On this I should like to mention two things. Of course, the money will be taken from our capital funds, and its distribution will for the moment lose us a very substantial amount of income, which has hitherto been available for our annual distributions. That will be a serious thing, but it is a problem we have to face, and we are optimistic enough to believe that we shall be able to face it and make good what I hope really will be only a temporary loss. The actual distribution, of course, will be a matter for the Distribution Committee. They have got a new problem to deal with. Hitherto they have always proceeded, as you know, on the basis of the audited accounts of the previous year. This, not being an annual distribution, but a distribution at a date when we cannot fall in with our ordinary formula, will no doubt require special handling, but we are quite satisfied that our Distribution Committee will be able to provide it, their knowledge and experience now being unrivalled in dealing with these problems.

### Further Special Efforts Essential.

Finally, I want to say this: this distribution is not going to be anything like a complete solution of the problem of the hospitals. In fact, we cannot, we are not expected to, finance the London hospitals, or even wipe out their existing debts off our own bat, so to speak; we can only supplement, firstly, the efforts made by the hospitals; secondly, the efforts made by kindred organisations; and, thirdly, and much more, the future efforts of the public to support these institutions, which are of untold value to the public. We hope that our example will be followed. We hope that the fact that the King Edward's Fund feels it necessary to make this very special effort will help to bring home to the public the fact that further special efforts are essential for the good of the country—not merely for the good of the hospitals themselves; and I need not say that I feel certain that everyone in this room will help us, or, rather, help the hospitals in bringing this cardinal fact home to the public; for, after all, it is with the public only that the future prosperity of our hospitals really rests. I do not think I need say anything further in moving the resolution. If it is carried we shall have made, I feel, a fine initial effort, and it remains for others to follow our example.

### The Hospitals Urgent Need.

Lord Revelstoke (Hon. Treasurer and Chairman of the Finance Committee), said that it was obvious that they would not be able to provide £250,000 without parting with some of their capital assets. It was equally evident that, although they would be able to provide this money by the sale of British Government securities having but a short term to run, the sale of even those securities would result in an appreciable loss, and would, of course, further result in a diminution of income.

The Speaker of the House of Commons said that before he could be sure that the assistance given by the Fund would be permanently effective in meeting the present emergency he would like to know what steps the hospitals themselves were prepared to take to stabilise their position.

Lord Burnham also referred to the necessity of adequate support being given by the working people of London. Their present contributions towards the maintenance of their hospitals amounted to only 10 per cent., as against 75 per cent. contributed by their fellows in some of the provincial towns to similar local institutions.

The resolution, having been also supported by the Earl of Bessborough (Chairman of the Executive Committee) and the Hon. Sir Arthur Stanley, was then put and carried unanimously.

### Message from the King.

Lord Stamfordham said that he had the honour to read the following message from His Majesty:—

"The King, Patron of the Fund, cordially approves the resolution passed by the Council of King Edward's Hospital Fund to make an emergency distribution of £250,000 as an earnest of their desire to do their immediate share in relieving the London hospitals of their difficulties. His Majesty trusts that other Societies and public bodies on whom the obligation in part rests, as well as private individuals, will join in this effort to meet the pressing needs of the London hospitals, and thus ensure the continuance of the voluntary system."

The proceedings then terminated.

